# Nursing students' experience of an alternative model for supervision during practical studies in the municipal health service: A qualitative study

**DOI:** 10.1016/j.heliyon.2023.e21719

**Published:** 2023-10-26

**Authors:** Therese Antonsen, Carita Stenberg, Kristin Hartveit Hansen, Beate André, Wenche Bergseth Bogsti

**Affiliations:** a Department of Public Health and Nursing, Norwegian University of Science and Technology (NTNU), 7491, Trondheim, Norway; b Department of Health Sciences in Gjøvik, Norwegian University of Science and Technology(NTNU), N-2802, Gjøvik, Norway

**Keywords:** Clinical practice, Nursing students, Peer learning, Reflection groups, Supervision, Qualitative methodology

## Abstract

**Aim:**

The aim of the study is to describe how Norwegian nursing students experience clinical practice when the Strengthened Supervision in Practice model is used together with peer learning.

**Background:**

Clinical practice is one of the most important parts of nursing education and the nurse supervisor plays an important role in the education of nursing students. Challenges arise because nursing students do not always receive quality supervision in practice. The quality of supervision affects the learning outcomes and well-being of the students during clinical practice. To meet the challenge that students do not always receive high-quality supervision, we wanted to try out a new supervision model Strengthened Supervision in Practice. Peer learning was also tried out in clinical practice.

**Method:**

The study used a qualitative design. Data were collected from three focus group interviews with a total of 11 nursing students participating.

**Findings:**

Clinical nurses are the most competent to supervise and assess nursing students in clinical practice. Peer learning provides safety in a learning situation.

**Conclusions:**

This study shows that the supervisor and their supervision competence are important for the student's learning. Cooperation with the lecturer in common meetings is important to make sure supervisors have quality guidance and assessment skills. The Strengthened Supervision in Practice model seems to meet expectations, but further research is necessary to develop the model further.

## 1 Background

Nursing students' well-being and achievement of learning outcomes in clinical practice are important for learning and training practical skills and for the recruitment of nurses in clinical practice [1]. It is well established that the clinical learning environment is sociocultural, and supervisors' relationships affect the general environment and students’ opinions of learning experiences [2,3]. A good learning environment for students in clinical practice is dependent on an explicit structure for receiving students, a pedagogical atmosphere where staff take an interest in the supervision of students and are easy to approach, and engagement among and collaboration between supervisors and lecturers [4].

Clinical practice is one of the most important parts of nursing education and the supervisor plays an important role in the education of nursing students [5,6]. A nursing home is a basic clinical practice field in nursing education [1].

As students in clinical practice, they can be taught tutored in new skills, and supervised in the practice of nursing [7]. Daily supervision is important for students learning [8]. Nursing students do not always receive quality supervision in clinical practice, and that is a big challenge in nursing education [9].

On this background the Universities need to offer optimal academic and supervision support, both to the students and their supervisors at the nursing home [1]. Earlier research has emphasized that improving nurse supervisors' abilities to supervise students is crucial to both improving nursing undergraduate education and ensuring that newly graduated nurses can successfully transition into nursing work in clinical practice [10].

Previous research has pointed out difficulties to identify and test the benefits of supervision models that improve learning in clinical practice in nursing homes for students [1]. More effective communication strategies should be established to ensure that nurse supervisors receive correct and enough information from the universities about student placement learning outcomes for clinical practice [11].

A reflection group with a mentor provides a deeper understanding and can stimulate critical thinking among nursing students [12,13]. Reflection may also help students to identify their learning requests and it can take place individually or at a group level [13,14]. Research has discovered that the incidents and the assessments of students in the first clinical practice are primarily expressed emotionally. Self-reflection may help students to express reactions and feelings to identify problems and, to understand themselves better [15]. If the students perform self-reflection and after that meet students at the same ward to reflect together, they take part in peer learning. Peer learning can be defined as the acquisition of knowledge and skills through active help and support among status equals or corresponding others [16]. Peer learning increases both self-confidence and competence and contributes to a reduction of “anxiety” in clinical practice [6,17]. To ensure the possibility of peer learning, practice units should accept at least two or more students from the same year in clinical placement.

Previous research has shown that the lecturers' support of students is important for the student's satisfaction with clinical practice [18]. The students also appreciated lecturers visiting them in clinical practice to supervise the student–nurse supervisor relationship and to provide personal support [19]. In Norway, students meet their lectures during clinical practice in discussions and assessment meetings together with the nurse supervisor. The supervisor and the students conduct the final assessment without a lecture. The lectures meet the students for group reflection during the clinical practice.

Training and supporting nurse supervisors is important [1,10], and based on this we have developed an alternative model for the supervision of students in clinical practice: Strengthened Supervision in Practice (SVIP) [8,20,21]. The main element of SVIP in this model is that nurse supervisors in clinical practice are allowed to develop their competence in supervision and increased responsibility for assessing the students. Nurse supervisors are responsible for professionally assessing the students' nursing achievement, and learning outcomes and their opinions are crucial in this overall assessment.

The role of the lectures from the university has changed in the SVIP model. The role has been transformed into being a facilitator, educator, and mentor to nurse supervisors. The lecturers has direct contact with the students during the reflection groups [8]. Reflection groups with the students and the lecturers are formalized through reflection groups during the clinical practice.

Traditionally the lecturer from the university have participated in meetings in clinical practice and had the responsibility for the assessment of the nursing students. In the new model (SVIP) nurse supervisors participate in three supervisors’ meetings with the lecturer during clinical practice. During these meetings, the nurse supervisors receive education from the lecturer to improve their daily supervision of the students. They also receive guidance from the lecturer in preparation to conduct the assessment meetings with the nursing students alone [20,22]. In this way the new model will increase the pedagogical competence for the nurse supervisor and strengthen the learning outcomes for the nursing students.

In summing up, the SVIP model in this project consists of supervision on three levels: (l) the nurse supervisor's supervision of the student, (ll) the lecturer educates and meets with supervisors, and (ll) the students' peer learning and participation in reflection groups. Based on this we will explore the following research question:

How do Norwegian nursing students experience clinical practice when the supervision model SVIP is used?

## 2 Methods

This study was conducted in the spring of 2021 among nursing students in their first year of the bachelor's program at the Norwegian University of Science and Technology (NTNU). Nurse education at NTNU takes place on three different campuses: Ålesund, Gjøvik, and Trondheim. This project was conducted at all campuses. This study was conducted by the COREQ checklist for qualitative studies [23].

### 2.1 Study design

Data collection was conducted using focus groups. A focus group interview is a qualitative method to get discussions and different points of view on themes in the study. We wanted to illuminate the informant's opinions and experiences and they together should discuss the advantages and disadvantages of the introduced model, and therefore focus groups were chosen [24].

### 2.2 Sample

An invitation e-mail was sent to all the students (N = 52) assigned to participate in the ongoing clinical research projects of introducing SVIP as a supervision model in clinical practice. The e-mail was sent at the end of the second semester and included information about the purpose of the study, topics for the focus-group interview, and how the time and place for the interviews should be agreed upon. Out of 52 eligible nursing students, 11 responded to the invitation. The student's ages ranged from 20 to 45 years old and were divided into three focus groups. All participating students consisted of 10 females and one male, as shown in Table 1. The students who participated were from all three campuses and had experience from practical placement in nursing homes in their first year of nursing education.

**Table 1:** Overview of cities, students, and nursing homes included in the study.

City	Total students at the nursing home	Total number of nursing homes included in the project
Trondheim	22	2
Ålesund	26	2
Gjøvik	4	1
	52 students in total	5 nursing homes in total

### 2.3 Data collection

The first, second, third, and last authors conducted three qualitative focus-group interviews [24]. All researchers were women holding a Ph.D. or Mc and were employed at the university. The researchers had different experiences in research, but both the fourth and last authors have extensive experience with both qualitative and quantitative research studies. A thematic interview guide with open-ended main questions such as*: “What is your experience of daily supervision from the supervisor?”* and “What is your *experience of group reflection, including your experience of peer learning?“*, followed by probing sub-questions (e.g., ‘could you give an example of’, ‘is there anything else you would like to add’) was used. The interviews lasted approximately 45 min and were conducted digitally because of the Covid-19 pandemic, who closed down all universities in Norway. Participants in the group interviews were gathered in groups from the same campus. This was done to facilitate a safe environment in the interview situation [24]. The interviews were conducted during the last weeks of the clinical practice period. All interviews were digitally recorded and transcribed verbatim by a professional transcriber service.

### 2.4 Data analyses

The analyses centered on meaning as explained in Ref. [24] to explore how nursing students experienced being supervised using the new model SVIP in clinical practice in nursing homes. The first author inductively read and coded all interviews. The research team had numerous physical and digital discussions to examine the content and descriptions of the codes, at some point in which several interpretations were established until a consensus of interpretation was achieved. The final analytical categories, presented in Table 2, were decided upon by comparing (finding matches) and contrasting (seeking for adverse cases) codes.

**Table 2:** Examples from the analysis process.

Central themes	Meaning categorization	Main categories	Subcategories	Quotation
Supervision	The role of the supervisors	The significance of supervision	*Supervision that promotes motivation* *Students' experience from assessment meetings* *Lecturer‵s task in clinical practice*	“My supervisor and I don't get along at all, I don't get feedback from her.” (Interview 3) “He is so very, very good at making you think for yourself, while he is also, well, good at making us think in the right direction.” (Interview 1)
Learning together	Peer learning	The significance of peer learning	Common growth through peer learning The importance of student peer learning to feel safe in the learning environment	“We can also learn from each other.” (Interview 3) “Going together in pairs has also made it much easier to become confident in your own skills.” (Interview 2)
Group reflection	Group reflection	The significance of group reflection	*Benefits from group reflection when listening to other students' clinical settings* *Learning to reflect on clinical settings*	“In a way, you also pay more attention to how important it is to reflect as well and talk about things you may be a little unsure about.” (Interview 2) “… get to hear others, other people's experiences, and yes, you learn from that too.” (Interview 1)

### 2.5 Ethical considerations

Interview participation was voluntary, and the informants received oral and written information about the purpose of the study and gave oral consent to participate. The Norwegian Centre for Research Data (NSD) approved the study (ref. no. 384451). Data were transcribed and anonymized accordingly. The publication of data was carried out following current research ethical guidelines [25].

Please note that our ethics requirements are now updated. Please choose all applicable statements in our ethics declarations list (available here: https://www.cell.com/heliyon/ethics) and include them as a complete ethics statement in the declarations section at the end of your manuscript.

### 2.6 Results

Three main categories to shed light on the aim of the study were identified through the analysis process, (see Table 3) [24]. The findings are further elaborated upon with statements from students, (further called informants), and the focus groups are numbered and given after every statement.

**Table 3:** Categories and subcategories.

Main categories	Subcategories
The significance of supervision	• Supervision that promotes motivation • Students' experience from assessment meetings • Lecturer's task in clinical practice
The significance of peer learning	• The importance of student interaction to feel safe in the learning environment
The significance of group reflection	• Benefits from group reflection when listening to other students' clinical settings • Learning to reflect on clinical settings

#### 2.6.1 The significance of supervision

The main category of the significance of *supervision* was created through the informants' sharing their experiences with having a supervisor and how supervisors influence the clinical practice.

#### 2.6.2 Supervision that promotes motivation

The informants were concerned about the supervisor's competence in supervision and how they use this competence to teach students to make their own decisions. They also emphasized the communication skills of the supervisors. The informants wanted those conversations with the supervisors to be characterized by openness in feedbacks about learning situations. One informant stated:

*“He is so very, very good at making you think for yourself, while he is also, well, good at making us think in the right direction.”* (Interview 1)

The informants were also concerned that they needed training in addition to feedback on how they could improve their skills in carry out nursing interventions. They pointed out that the supervisors gave them skilled confirmation and supervision on what could have been done differently:

*“He’s very direct, he tells us when we do things well and tells us when we do things we should not have done, in a way, or like that … should have done better. It’s very concrete and very easy to learn, in a way. And then there is room for error*.*”* (Interview 1)

The informants stated that they received supervision and feedback on their work in clinical practice this led to them feeling confident and more motivated to develop their skills. This kind of supervision made them feel more secure in their role as a student. Some also explained they were motivated by feedback from their supervisors. One informant described the positive effect of regular feedback:

*For my part, at least, I feel that I have become more confident, about what it is I should do and what I should not do, in a way. Like before, when I have been an assistant and stuff, I have been very insecure and just did what I thought, but now it's like that, now I base the choices I make on knowledge instead.”* (Interview 1)

The informants' discussion emphasized the importance of obtaining a form of competence confirmation and highlighted the potential for improvement among the supervisors.

*“I became a little more confident in the role as well, I think. So that we are students, that in a way it is allowed to ask about things that are a bit obvious, and yes, that in a way you are allowed to … have room to learn.”* (Interview 1)

The informants were concerned with being appreciated in their role as students in the unite and those who were supervisors were interested in been in the roll as supervisor. A story was told about asupervisor who was not interested in being a supervisor, and the informant felt like a burden, and this led to the students felt like a burden to the supervisor and were not valued as a student to practice and learn nursing intervention. When the informants did not feel valued, they became insecure in their student role and their motivation for the clinical practice was negatively affected.

#### 2.6.3 Student's experience from assessment meeting

The informants were concerned with targeted supervision so that they were confident that they had achieved the criteria for passing practice. They had different experiences if the supervisors were prepared and knew documents for assessment that were valid for conducting mid- and final assessments, as stated:

*“At least my supervisor was very prepared. She had documents ready … … then she helped me develop further, so I think it has gone very well.”* (Interview 3)

Other informants had the opposite experience:

*it seemed like the supervisor was a bit unsure of how to use the documents. She did not quite understand.”* (Interview 2)

The lecture was not present in the assessment meetings for the clinical practice in this project. To be able to assess the performance of nursing interventions, it is important that to be present in clinical everyday life to be able to observe the student. This gives the students reassurance that the assessments are based on the development and learning of nursing interventions during the clinical practice. One of the informants said:

*“For my part, it was very good because I feel that it is the supervisor who has seen how I have worked and how I have developed, and what goals I have reached. So, I think it went very well without a nursing Lecture.”* (Interview 3)

#### 2.6.4 Lecturer's task in clinical practice

In a clinical practice where there is a challenging relationship between supervisor and student, the lecturer in the assessment meetings can be a neutral person and bring a sense of safety to the student. The lecturer then also becomes a witness to what happens in the assessment meetings and can follow up with both the supervisor and the student.

*I think it could have been a lecture present then, just for directing that conversation - directing the feedback and stuff like that. Because with me, it just felt like an eternal critique. And I feel that, if the lecture had been there then it would have been a little more formalized "This you did well, this you did badly." … it depends very much on the relationship with the clinical nurse*. (Interviews 3)

#### 2.6.5 The significance of peer learning

The informants were asked about their experience of peer learning, and the informants expresses positive experiences with the use of peer learning.

#### 2.6.6 Common growth through peer learning

The findings indicate that having a peer student in clinical practice, they felt more confident in student role. Through peer learning, the informants prepared together before the learning situation. They asked each other questions, completed nursing interventions together, and reflected after each learning situation. One of the informants described their experiences:

*I think it has been very reassuring to have someone you are paired with. Because it's a bit like this when you … yes, none of us had any special experience, and the fact that you have each other to rely on, only the part of the reflection after you have been in a room to take care of a patient, then it's a bit difficult to talk to each other because you have to be with the patient, so then it is better that you can talk about it afterward*. (Interview 2)

The experience of peer learning depended on the supervisor's ability to follow up on a learning situation. When peer learning was used the informants experienced working independently, without a supervisor.

*Now we go quite independently a bit like that in the room and stuff, but we always have a brief then, or like that … we just call it that. Now we have a brief. Like, talking about, if it was something difficult, about how it went, yes, if we had any questions, and we have that every day in practice. He always asks if we have any questions and then asks if we need help before we go into different rooms and stuff*. (Interview 1)

#### 2.6.7 The importance of student peer learning to feel safe in the learning environment

By introducing peer learning in clinical practice, the student experienced this positively in relation to having an equal discussion partner. By using peer learning, the students can supervise each other in nursing interventions. The informants explained that peer learning influenced their experience of being a student in practice.

*Yes, well, it would have been a completely different experience if I had gone alone because we have in a way supported each other very much, and in a way, if one remembers something, then … eh, yes, and when taking care of a patient we help each other and are very proud of each other, so it has worked great. I'm very happy about that.* (Interviews 2)

#### 2.6.8 The significance of group reflection

The main category *Group reflection* emerged as a topic in the interviews through the fact that experiences with group reflection were requested.

Benefits from group reflection when listening to other students’ clinical experiences.

The informants described their experiences with presenting histories from clinical practice to other students in the reflection group and receiving feedback on what was presented. Learning situations from clinical practice were the source for group reflection. Through sharing stories from clinical practice and learning learn from each other student's description of failure and achievement. This created safety and motivation in clinical practice. The informants searched for acceptance of how to conduct nursing interventions in a correct way in clinical practice

*“To get confirmation then, that it was not a stupid way to handle the situation. Yes, things like that.”* (Interview 1)

During group reflection, the informants received new ideas and measures they could use during the clinical practice. When a student told their story from the clinical setting, the others in the group reflected on and learned from it.

#### 2.6.9 Learning to reflect in clinical practice

When nursing students are in clinical practice, they must learn to reflect on activities and interventions so that they can learn to be aware of unlike approaches to challenges in clinical practice. The informants described that reflection on nursing intervention was difficult and required practice and experience, this provided in reflection groups. Another benefit of group reflection they described was to become aware of how important it was to reflect on clinical settings.

*“But I think it was very good to have such a day of reflection, because then in a way you also became a little more aware of how important it is to reflect as well and talk about things you might be a little unsure about or, yes, wondered about like, "Oh, was this right, wasn't it?" Yes.”* (Interview 2)

## 3 Discussion

The aim of this project was to examine *how Norwegian nursing students experience clinical practice when the supervision model SVIP and peer learning are being used.*

### 3.1 The experience of supervision

The informants in this study appreciated getting feedback and confirmation of their performance in learning situations in clinical practice, which is in line with previous research [8]. The informants stated that when the nurse supervisors gave them feedback before and after their performance in a learning situation, it gave them a sense of safety which was experienced as a positive.

Previous research shows that SVIP can lead to supervisors taking a closer look at the criteria for the learning situations [20]. In this current study, the informants expressed that the nurse supervisor had different knowledge of the assessment documents with learning outcomes. Nevertheless, the informants pointed out that it was positive the supervisor conducted the formal assessment meetings without the lecturer present, as the nurse supervisor was the one who observed them in the clinical practice. The informants in this study stated that the nurse supervisors had the necessary knowledge to make the assessment. Earlier finding suggests that nurse supervisors, through the supervisor meetings, gain increased knowledge about the learning outcomes and how these can be used in everyday clinical life and assessment situations [20].

Findings in this study suggest that there is no need to have a lecture presentation in the assessment meetings if a good relationship with the nurse supervisor is established, as earlier research also has found [8]. However, our findings showed that some informants described problems and negative experiences with their nurse supervisors. In these situations, it would have been positive to have the lecturers present to participate in the dialogue and be a “witness” to what happens in the meetings. The lecture may contribute to more structure in the assessment meetings [8]. Price, Hastie [19] research shows that nursing students value the lecturer's role in the supervision of the student-nurse relationship. Other research shows that nursing students had expectations that the lecturer would act as their spokesperson if they needed such support [26]. Lecturers' support of students is important for the student's satisfaction with clinical practice [18].

### 3.2 Peer learning

This current study suggests that the informants reported becoming more confident in skills with peer learning. They had a common experience of a learning situation and had a common basis for discussing and reflecting on their nursing practice. Peer learning is a useful method that improves student self-efficacy [27]. Findings in our study revealed that the informants enjoyed being in learning situations with supervisors and other students. Informants in our studies who reported they had not received feedback and supervision in conjunction with learning situations were not satisfied with the supervision they received. It can be difficult for students to report that they want supervision because practice also is an assessment situation [8]. Peer learning can be stressful and frustrating for some students [28,29].

Supervisors' knowledge of peer learning was important for the implementation of peer learning [28]. In the supervisors’ meetings, it can be a focus area to increase competence about peer learning; the supervisors contribute to how it can be carried out in practice. Other studies have reported that supervisors feel they can relinquish control and take a step back to let the students carry out learning situations alone when peer learning is used [29,30]. Both supervisors and students experienced that peer learning promotes the learning process [30]. Our findings show that peer learning provides safety in a learning situation, which indicates the implementation of SVIP with peer learning is successful for nursing students in clinical practice.

### 3.3 Group reflection

The informants in our study participated in group reflections, where they learned from each other by presenting narratives from practice and received support and feedback on how they had managed the situations. They had the opportunity to reflect on their actions and hear others' reflections on their actions. Research shows that reflection stimulates learning in practice [13] and students learn from each other [31]. The findings highlighted that they learned to reflect through the reflection groups, and the reflection group can stimulate reflection.

Group reflection is a meeting point for lectures and students when the students are in clinical practice. A reflection group with a mentor provides a deeper understanding and can stimulate critical thinking among nursing students [12,13]. The degree of preparedness among the students for group reflection influenced the outcome for every student. The amount of satisfaction with the lecture in practice was directly linked to the number of meetings held between the student and the lecture [32]. The informants in this study recommend that there should be more reflection groups during a clinical practice period.

## 4 Methodological considerations

The qualitative method is appropriate for bringing out the informants' experiences. We consider focus groups as an appropriate method for focusing on diversity and nuances in the informants’ experience of the implementation of the SVIP model.

Most of the informants involved were female, which reflects that most students in nursing education are female. The informants were divided into focus groups depending on which campus they belonged to. This may have established a safe space to share knowledge but may also have led to informants not being open about challenges with their peer students.

The interviews were held digitally, which may have been a barrier to dialogue between the informants; this is important in a focus group. In contrast, conducting the focus group digitally may have created a safe space as there is a distance from the other informants. Due to the low number of informants, these findings cannot be easily generalized.

## 5 Conclusion

The aim of the study was to describe how Norwegian nursing students experience clinical practice when the Strengthened Supervision in Practice model was used together with peer learning. Peer learning was highlighted by the nursing students as important for learning and well-being in clinical practice. Group reflection with the lectures can lead to an experience of how important it was to reflect on clinical settings. This study shows that the supervisor and their supervision competence are important for the nursing student's learning. The nursing students had positive experiences with the supervisor conducting the formal meetings as the supervisors had the daily supervision in clinical practice. The SVIP model seems to have improved the quality of supervision in clinical practice for nursing students, but further research is necessary to develop the model further.

## Funding sources

Norwegian Directorate for Higher Education and Skills, PILOT-2019/10005. They have no role in conducting the research.

## Data availability statement

Data will be made available on request.

## Credit authorship contribution statement

**Therese Antonsen:** Writing – review & editing, Writing – original draft, Software, Methodology, Investigation, Formal analysis. **Carita Stenberg:** Writing – review & editing, Methodology, Investigation, Formal analysis. **Kristin Hartveit Hansen:** Writing – review & editing, Methodology, Investigation. **Beate André:** Writing – review & editing, Supervision, Methodology, Investigation. **Wenche Bergseth Bogsti:** Writing – review & editing, Supervision, Methodology, Investigation, Funding acquisition.

## Declaration of competing interest

The authors declare that they have no known competing financial interests or personal relationships that could have appeared to influence the work reported in this paper.
