# Supplementary material for: Nursing students' experience of an alternative model for supervision during practical studies in the municipal health service: A qualitative study
Source: Heliyon. 2023 Oct 26;9(11):e21719. doi: 10.1016/j.heliyon.2023.e21719 (PMC10643253; doi:10.1016/j.heliyon.2023.e21719)
Supplement: Multimedia component 3 [file mmc3.pdf]

INTERVIEW GUIDE – NURSING STUDENTS

Introduction

- Inform about the study in general and the purpose of the interview.
- Inform that the interview will be recorded and that the transcripts will be available to researchers in the project.
- Reminder that the informant can withdraw his consent at any time, without reason.

Background information

• Experiences

• Education

Theme 1 – Experience of supervision and assessment during the clinical practice

• How did you experience the daily supervision from the supervisor?

• How did you experience your own learning in relation to the learning outcomes during the clinical practice?

• What was the expectation of the clinical practice? What was good, and what was challenging during the clinical practice?

Theme 2 – Reflection groups

• How did you experience the reflection groups?

• How was peer learning facilitated during the reflection groups?

• What are you left with after the reflection groups?

Theme 3 – Conclusion

• Is there anything else in relation to guidance from the practice supervisor or contact teacher that has not been highlighted and that you would like to address?
